# Efficiency and Complications of Esophageal Stenting in the Management of Postoperative Fistulas

**DOI:** 10.3390/jcm13206167

**Published:** 2024-10-16

**Authors:** Cristian Gelu Rosianu, Andreea Pușcașu, Petre Hoara, Dragos Predescu, Rodica Birla, Florin Achim, Vlad Codrut Strimbu, Silviu Constantinoiu, Octavian Andronic, Alexandru Constantinescu

**Affiliations:** 1 Department of General Surgery, “Carol Davila” University of Medicine and Pharmacy, 050474 Bucharest, Romania; gelu-cristian.rosianu@drd.umfcd.ro (C.G.R.); petre.hoara@umfcd.ro (P.H.); dragos.predescu@umfcd.ro (D.P.); rodica.birla@umfcd.ro (R.B.); florin.achim@umfcd.ro (F.A.); silviu.constantinoiu@umfcd.ro (S.C.); 2 Gastroenterology Department, “Saint Mary” Clinical Hospital, 011172 Bucharest, Romania; 3 Faculty of Medicine, “Carol Davila” University of Medicine and Pharmacy, 020021 Bucharest, Romania; andreea.puscasu@stud.umfcd.ro; 4 Centre of Excellence in Esophageal Surgery, “Saint Mary” Clinical Hospital, 011172 Bucharest, Romania; 5 Center of Innovation and e-Health, “Carol Davila” University of Medicine and Pharmacy, 020021 Bucharest, Romania; vlad-codrut.strimbu@rez.umfcd.ro; 6 Nephrology Hospital Dr. Carol Davila, 010731 Bucharest, Romania; 7 Department of Gastroenterology, “Carol Davila” University of Medicine and Pharmacy, 050474 Bucharest, Romania; alexandru.constantinescu@umfcd.ro

**Keywords:** esophagus, stent efficiency, anastomotic fistula, esophageal cancer, fistula closure

## Abstract

Esophageal anastomotic fistula (AF) is a frequent and severe complication of an esophagectomy due to esophageal or eso-gastric junction cancer, regardless of the selected surgical technique. AF is usually treated by endoscopic stent placement. **Objectives**: This study aims to examine the efficacy of stents in the treatment of AF, analyzing the healing period and the factors that contribute to its delay. **Methods**: We collected data from 55 patients who underwent stent implantation for AF, and analyzed multiple variables related to patient healing time and surgical technique with two primary endpoints: post-stenting hospital stay and the time of stent usage until fistula closure. The patients were divided into three groups based on the anastomosis type (eso-gastric anastomosis, eso-gastric cervical anastomosis and eso-jejunal anastomosis) and they were compared using the primary endpoints. **Results**: Our findings show the differences between the three groups, with a longer hospital stay for eso-gastric anastomosis, and an extended time of fistula closure in the case of eso-gastric cervical anastomosis. We also found a significant correlation between the size of the fistula and the hospital stay (R = 0.4, *p* < 0.01). Regarding patients’ risk factors, our results show an extended post-stenting hospital stay for those patients that underwent preoperative radiotherapy. **Conclusions**: Our results offer an extended view of the efficiency, hospitalization duration and healing time for esophageal anastomotic fistula, and reveal some of the factors that interfere with its resolution.

## 1. Introduction

Esophagectomy with esophageal reconstruction is a complex procedure, serving as the gold standard for treating esophageal or eso-gastric junction cancer [1]. Regardless of the selected surgical technique, anastomotic complications are found in 25–70% of cases [1,2,3,4,5] due to the lack of a serosal layer and the vertical orientation of the muscular fibers that provide poor support for the sutures and staples [6]. The most common complications are anastomotic leaks and stricture formation [7], but the most alarming and life-threatening one is anastomotic fistula [8], having a postoperative incidence rate between 3% and 25% [9,10,11]. Surgical complications, such as stapler misfire or over-tensioned sutures, can lead to anastomotic fistulae, but also common causes such as local hematoma, low perfusion, or tissue trauma are involved [12,13]. The anastomotic fistula (AF) can communicate with the respiratory tract (airway-gastric fistula—AGF) [14] or with the large vessels in contact with the esophagus (aorto-esophageal fistula) [15], with an increased prevalence of intracervical anastomosis compared to intrathoracic location [16].

Evaluating the preoperative risk factors, the medical literature presents a greater rate of anastomotic complications for elderly patients, serum albumin levels lower than 3 g/dL, a diabetes mellitus diagnosis and poor nutritional status [17,18,19]. Preoperative radiotherapy has shown controversial results related to anastomotic complications of the esophagus; some studies confirm a statistical correlation [20], but others do not [21].

The clinical presentation of anastomotic fistulae is heterogeneous and involves a cough, chest pain, recurrent pneumonia, severe hemoptysis, sepsis or respiratory failure in the AGF [22], epigastralgy, and hypovolemic shock in the aorto-esophageal fistula [23]. Clinical suspicion must be confirmed by esophagography [24], endoscopy with optional bronchoscopy and gastroscopy [14] or computer tomography scan with an oral contrast substance [10]. Other studies proposed diagnostic methods such as pleural drain amylase levels and the NUn score based on serum albumin, C-Reactive Protein and white cell count, but are still insufficiently researched [25].

The management of esophageal anastomotic fistulae involves an initial conservative treatment by thoracic drainage, fistula tube drainage, intestinal nutrition, and antibiotics [26]. Despite that, curative treatment requires procedures that use stents, endoscopic clips, endoscopic vacuum-assisted closure, or surgical methods such as primary closure and vascularized pedicle tissue flaps [27]. The systematic review of Schaheen et al., shows that the optimal management of esophageal anastomotic fistulas is the placement of stents, with leak resolution in 72% of cases [28]. The esophageal stents can be partially or completely covered, the chosen material being plastic or metal. Previous studies do not show statistical differences between stents in the postoperative success rate, being 84% for self-expanding plastic stents, 85% in the case of fully covered, self-expanding metal stents, and 86% in partially covered, self-expanding stents [29]. However, the disadvantages of using stents are the prolonged closure time that varies between 1 and 197 days, and stent complications such as migration, rupture, tissue ingrowth or overgrowth, severe pain, rupture of the esophagus, and hemorrhage–events that occurred in 46% of cases [30,31].

Even if the esophageal stenting procedure and its complications are commonly studied, the medical literature has cited only a few articles that address the specific issue of AF [14,25,32,33,34]. Moreover, there are a lack of data on the anastomotic fistulae outcomes and the efficiency of esophageal stenting on AF resolution. Only three articles focus on the endoscopic treatment of the AF after esophageal or eso-gastric junction cancer, including patient groups of 35 [14], 25 [32] and 5 [30]. These studies focus only on the risk factors that predispose patients to AF and the efficiency of stenting for anastomotic closure, but they lack aspects that interfere with the healing time. Therefore, further studies on a wider patient group should address the issue of fistulae treatment in order to maximize its efficiency and accelerate the healing time.

## 2. Materials and Methods

This study was a retrospective single-center study with patients from the “Saint Mary” Clinical Hospital, Bucharest, Romania. We collected patient data from January 2020 to January 2024, with all the patients that had a postoperative esophageal anastomosis fistula being included.

### 2.1. Endoscopic Procedure

**Figure 1:**
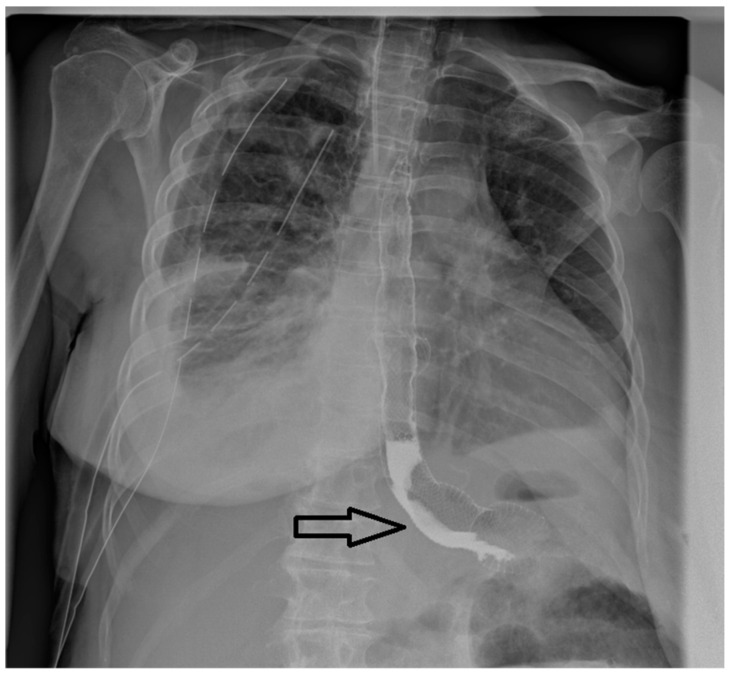
Radiological appearance after deployment of the esophageal stent within an eso-jejunal anastomotic fistula after the surgical resection of a gastric neoplasm.

Upper gastrointestinal endoscopies were conducted under general anesthesia, with orotracheal intubation and X-ray guidance (Figure 1). 

**Figure 2:**
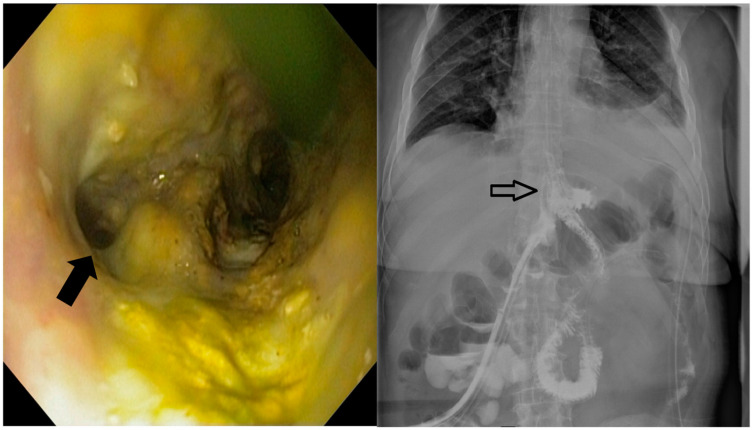
The endoscopic appearance of an eso-jejunal anastomotic fistula following surgical resection of a gastric neoplasm, along with the radiological findings after oral contrast administration.

These procedures were performed by interventional endoscopists using an endoscope equipped with a CO_2_ inflator. During the diagnostic phase, the fistula (Figure 2) was confirmed and characterized through endoscopic visualization and contrast opacification under fluoroscopy. Key aspects assessed included the size of the orifice (<1 cm, 1–2 cm, >2 cm), the condition of the edges (whether necrotic, inflammatory or fibrous), the presence of pus and any drainage. Based on these findings, the therapeutic phase involved placing a covered metallic stent, applying a clip or using a combination of treatments. The specific endoscopic devices used, and the absence of residual leakage after contrast injection at the end of the procedure, were documented.

**Figure 3:**
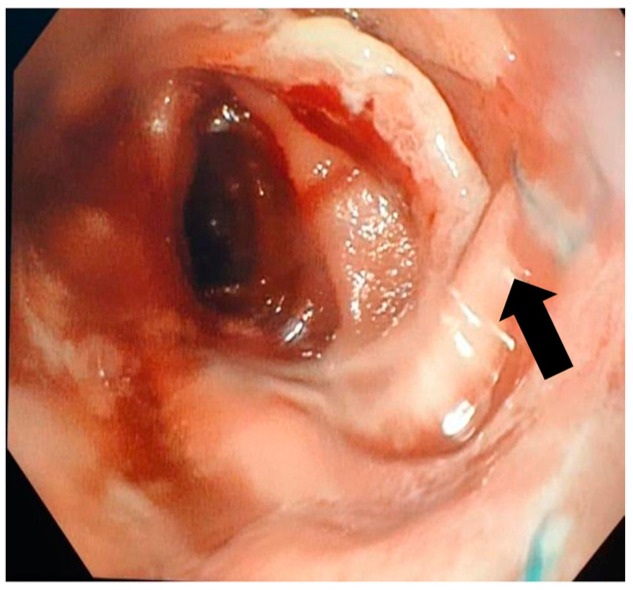
Eso-jejunal anastomosis after stent extraction with closed fistula after the resection of a gastric neoplasm.

A follow-up endoscopy was routinely performed within 6–8 weeks, provided no adverse events occurred, to remove the stent, assess the treatment’s effectiveness and determine if additional intervention was needed (Figure 3). During this and any subsequent endoscopies, the migration and removability of the stents were evaluated, along with the persistence of the leak following the contrast injection (after the removal of the stent and/or any remaining clips). Endoscopic treatment was repeated until either the treatment was deemed effective, surgical intervention was required or the patient succumbed.

### 2.2. Patient and Procedure Variables

For each patient, we collected information about the patients and their biological status, such as sex, age, height, weight, smoking status, alcohol consumption status, blood pressure, heart rate, oxygen saturation, hemoglobin, serum albumin, glycaemia, serum creatinine and comorbidities; about the tumor, such as tumor location, the presence of preoperatory dysphagia, tumor dimension, histopathological tumor diagnostic, TNM and grading classification; and about the presurgical, operative and postoperative information, such as the surgery type, the presence of preoperative radiotherapy or chemotherapy, the intent of the surgery, the tumoral presence in the surgical margins, the location of the fistula, the imagistic method of fistula detection, the primary stent type, the postoperative hospital stay, the presence of jejunostomy and its usage, postoperative survival, cause of death, the number of stents used, the usage of a second stent, the presence of mediastinitis or of peritonitis, the pathogen detected in mediastinitis or peritonitis, the presence of an esophageal–pleural fistula, stent complications and their treatment, the time period until complication diagnostic, the successful closure of the fistula, the time period of the postoperative fistula diagnostic, the endoscopic dimension of the fistula and the time period of stent usage until fistula closure. In order to maximize the information from our lot, we decided to use the fistula location as the variable in our analysis instead of surgical technique as the two are equivalent. Using the anastomosis type, we divided the lot into three groups of study when analyzing patient healing time and hospitalization duration: the patients with eso-gastric anastomosis, eso-gastric cervical anastomosis and eso-jejunal anastomosis.

### 2.3. Statistical Analysis

The mentioned variables were analyzed using R Statistical Software [35]. In order to evaluate relationships related to categorical variables, we used the Chi-square test, or more often the Fisher exact test (with patients per category below 5). In the case of numerical variables, we used the correlation coefficient and regression. In order to assess the differences between different treatment regimens, we used the F test followed by Student’s *t*-test with a Bonferroni correction to determine the pair differences. For comparisons between patient groups, we used two-sample *t*-tests. All tests used the standard statistical significance of *p* < 0.05. We used two primary endpoints for comparing surgical technique/anastomosis location: post-stenting hospital stay and the time of stent usage until fistula closure.

## 3. Results

### 3.1. Patient Characteristics and Surgical Outcomes

Our study included a total of 55 patients between the ages of 41 and 80 years old who were operated on at the University Emergency Hospital, Bucharest, for esophageal or eso-gastric junction cancer during the evaluation period (Table 1). There were 30 patients that had radiotherapy before surgery and 50 had chemotherapy. In terms of the tumoral cell type, 46 patients had adenocarcinoma and 9 had squamous cell carcinoma. Of these 17 were stage II, 34 were in stage III and 4 were in stage IV. Tumor size ranged from 2 to 4 cm.

**Figure 4:**
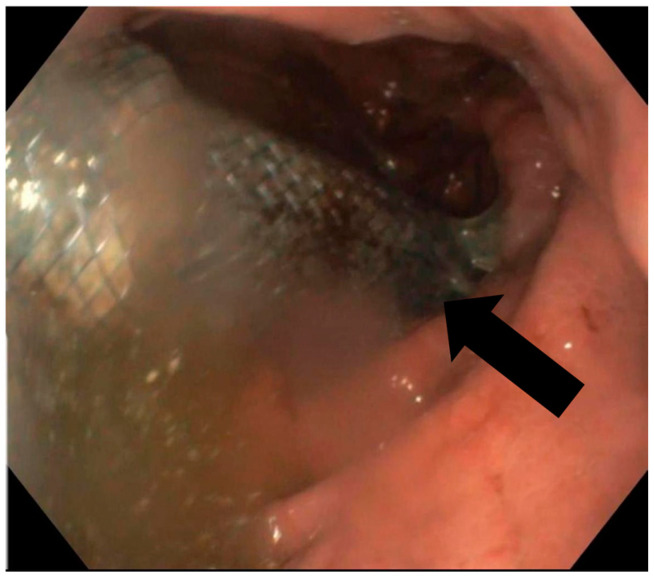
Endoscopic appearance of the stent migrated to the gastric graft after Ivor Lewis esophagectomy.

**Figure 5:**
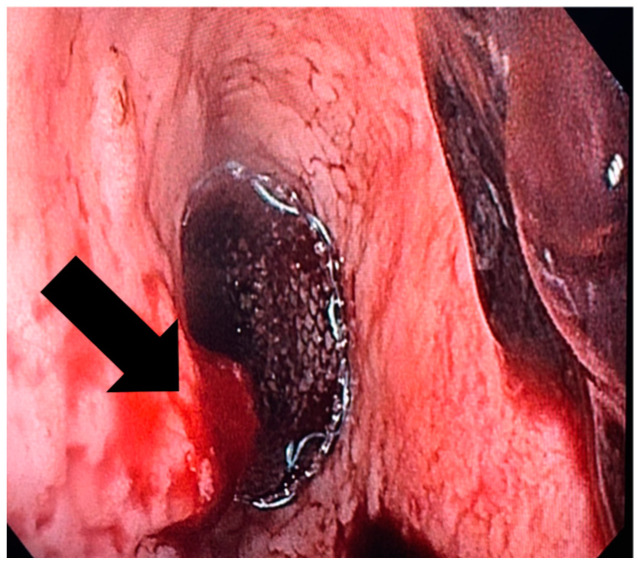
Endoscopic appearance of an esophageal stent-induced hemorrhage at the esophageal end of the stent inserted for the closure of an eso-jejunal anastomotic fistula.

In terms of the surgical outcomes, three surgical techniques were used on most of the patients (Table 2): Ivor Lewis (17 patients) with eso-gastric anastomosis, McKeown (4 patients) with eso-cervical gastric anastomosis, and total gastrectomy with esophago-jejunostomy (34 patients). There was one patient who had also a partial hepatectomy due to tumoral invasion in the liver, and one who had a partial gastrectomy. In our study, only complete cover stents were used, with four stent sizes. The average period for stent usage was 32.4 days with an average period of 7.4 days post-surgery at which a fistula was diagnosed. We have to mention that there were no patients that had immediate post-surgical leakage present at the methylene blue leak testing. The average hospital stay by anastomosis type was 19.1 days for eso-gastric anastomosis, 4.5 days for the eso-gastric cervical anastomosis, and 8.9 for the eso-jejunal anastomosis. Patient survival was 95%, with a total death count of three. The fistula closure rate was 98% (54 patients), with 2 patients (out of 54) not surviving due to later complications. However, post-stenting complications were more often present, with a rate of 20% (11 patients). Most often the complications were stent migration (Figure 4), stent-induced hemorrhage (Figure 5) and stent perforation. There were 12 cases of post-stenting mediastinitis, out of which 2 resulted in patient death. Another death was due to an aortic-esophagus fistula.

### 3.2. Statistical Analysis of Patients Data

When using our primary endpoint analysis, it was found that there was a difference between the three treatment groups in terms of both hospital stay and stent usage until fistula closure. For hospital stay, our pairwise comparison revealed that the eso-gastric anastomosis was statistically different from the other two (*p*-value < 0.01). However, eso-jejunal and eso-gastric cervical anastomoses did not show a significant difference (*p*-value = 0.29). For stent usage until fistula closure, we found that only eso-jejunal anastomosis was different from the eso-gastric cervical one (*p*-value = 0.03), with the eso-jejunal anastomosis having a longer stent usage (6.06 days) until fistula closure.

We performed multiple comparisons for the above-mentioned variable. We found that there was a significant correlation between the size of the fistula and the hospital stay (R = 0.4, *p* < 0.01). In addition, the regression analysis revealed a slope coefficient of 0.86, which can be interpreted as: every increase in fistula size with 0.86 mm extends the hospital stay by 1 day. We also found a significant difference in hospital stay between patients who received radiotherapy, and those who did not (*p* = 0.01). This result was not seen in those who received chemotherapy. 

In addition to the significant differences found, we obtained multiple negative results that we considered important to mention as well, as they represent important findings, and also we wanted to avoid positive publishing bias [36]. There were no differences between the anastomosis locations in terms of patient mortality (*p*-value = 0.4) or surgical complications (*p*-value = 0.8). We found no difference in terms of complications between the stent sizes (*p*-value = 0.12). There were also no significant correlations between hospital stay and serum albumin (*p*-value = 0.06), hemoglobin value (*p*-value =0.1) or serum glycemia (*p*-value = 0.09). The negative findings have to be interpreted cautiously, considering the limitations of our data.

## 4. Discussion

The medical literature has studied the topic of endoscopic treatment for esophageal anastomotic fistula mostly regarding the efficiency of stenting on fistula closure. Therefore, we decided to focus our study on the factors that can influence the healing time, enabling the surgeon to obtain a greater perspective on patient’s prognosis. Our primary outcome was to analyze the relation between the anastomotic location and the parameters related to the healing period. Secondly, we wanted to explore other factors that interfered with the resolution of the esophageal anastomotic fistula.

We investigated the effectiveness of stenting for AF through two parameters related to the post-stenting healing period: the post-stenting hospitalization time and stent usage until fistula closure. 

Firstly, we will discuss the implications of post-stenting hospitalization time. In our patient group, the average period of hospital stay is 11 days, a result in accordance with other studies on this topic that revealed an average period of 6–11 days [37]. By further implying the anastomotic location, we discovered that the hospitalization time for eso-gastric anastomosis was statistically longer from the other two (*p* < 0.01), with an average period of 19.1 days. The studies of Gonzalez et al. [38], and Blewett et al. [39], did not report any significant difference between the different anastomotic locations regarded in terms of hospitalization time. The delayed resolution of AF in the case of eso-gastric anastomosis could guide the surgeon in choosing a more beneficial operative technique for the patient, but more extensive studies must be carried out in this direction.

Secondly, the medical literature describes the average time to achieve the success of endoscopic treatment in AF as 23–44 days [31,38,40], confirming our results of a mean period of 32.4 days. We advanced the study by analyzing the difference between stent usage until fistula closure and the anastomotic location, and we found that only eso-jejunal anastomosis was different from eso-gastric cervical anastomosis (*p* = 0.03), requiring a longer stent usage until fistula closure. This result could represent an argument against eso-jejunal anastomosis in the process of choosing the ideal surgical technique for esophageal or eso-gastric junction cancer, but we have to consider that this reported time period can be influenced by other factors such as patients’ comorbid conditions or lifestyle factors.

Studying the association between the diameter of fistulae and other parameters in relation to the efficiency of stenting, we found a significant correlation between the AF size and the post-stenting hospital stay (R = 0.4, *p* < 0.01). There was an extension in the duration of hospitalization by one day for each increase of 0.88 mm in the AF diameter. This relation has not been documented before in the medical literature, even if the diameter of the fistula is an important aspect to consider when predicting the evolution of esophageal anastomosis healing. There is one study that shows the impact of fistulous orifice size on stenting efficiency, but it compares its diameter to primary and secondary stent closure [38]. Therefore, this unexplored approach proposes a more accurate correlation between the AF diameter and the patient prognosis, enabling the physician to obtain an ample view of every patient’s healing evolution.

Regarding the anastomosis location, we found no relevant statistical differentiations in terms of mortality (*p* = 0.4) or the risk of post-stenting complications (*p* = 0.8). Our results are similar to the study of Blewett et al. that illustrated similar leak incidence rates for cervical and intrathoracic anastomosis [39]. However, other studies attested to the superiority of intrathoracic anastomosis in better outcomes, explained by the increased anastomotic tension and scarce vascularization found in cervical anastomosis [41].

Individual risk factors of patients, such as hypoalbuminemia, anemia, serum glycemia or presurgical radiotherapy, and chemotherapy should also be addressed as a predictor for stenting efficiency. Firstly, the medical literature reveals a correlation between hypoalbuminemia and the occurrence of AF after esophagectomy [17]. In our study, only 31% of patients were diagnosed with hypoalbuminemia and no significant correlation could be realized between serum albumin and hospital stay (*p* = 0.06). Regarding anemia, our results correspond to the study of Patil et al., showing no influence on leakage rate for hemoglobin below 10 mg/dL (*p* = 0.1) [18]. Our patient group presented hyperglycemia in only 15% of cases and no significant correlation with the hospitalization period (*p* = 0.09), which could not establish diabetes as a risk factor for AF [16] due to the low number of patients. 

Preoperative radiotherapy’s influence on AF is a questionable subject in the medical literature, due to the differences between the studied groups. Even if many studies show no impact of radiation on leakage rate [42,43], we conclude that 55% of our patients diagnosed with AF underwent presurgical radiotherapy. Moreover, we found a significant difference between patients who experienced radiotherapy and those who did not in terms of the post-stenting hospital stay (*p* = 0.01). One possible explanation for this could be the degrading effect of radiotherapy on the tumoral and surrounding tissue which can result in worse surgical outcomes. This finding could influence the surgeon in choosing the advantageous therapeutic approach, keeping in mind the risk of prolonged hospitalization, and healing time for applying preoperative radiotherapy. Such differentiations were not observed for chemotherapy treatment, a result in accordance with the study of Doty et al. that shows no impact of preoperative chemotherapy on the AF healing process [44].

Stenting as an efficient means of AF resolution had favorable results in our patient group, showing a primary efficiency of 90% after the first stent placement and a secondary efficiency of 98%, after the second stent, having an overall survival rate of 95%. These findings exceed the results cited in the medical literature, where primary and secondary efficiency was defined as 50% and 70%, respectively [31,38], proving the superiority and efficiency of endoscopic stenting as a treatment for AF. In addition, the post-stenting complication rate in our study was 20% (11 patients), considerably decreased compared to an average complication rate of 46% in the study of Fabbi et al. [10], which may be due to a different selection of the patients that should undergo this procedure. Among the most common complications of the stenting procedure, we found migration in three patients, stent-induced hemorrhage in six patients and stent perforation in two patients. Analyzing the risk of the migration associated with a small-sized stent, we concluded the absence of statistical correlation between the stent dimension and the higher risk of post-stenting complication (*p* = 0.12).

Our study is associated with some limitations, such as the limited group of patients and the single-center research. Also, these results have to be interpreted with caution, as there were only three deaths in our patient group. More research with larger patient groups that include higher mortality rates is required for a better comparison in terms of survival, especially considering the fact that there were only four patients in the eso-gastric cervical group. Another limitation of the study is the lack of data related to the patient’s medical history and lifestyle factors that could interfere with the healing period of the AF that we omitted during the statistical analysis.

## 5. Conclusions

Endoscopic stenting for anastomotic fistula, a complication of esophageal or eso-gastric junction cancer, is an effective therapeutic approach with a great efficiency rate. Our study evaluated the outcome of stent placement by analyzing the healing time and stent usage until fistula closure regarding selected surgical techniques, anastomotic location, fistula size and other individual risk factors. These findings could offer an ample perspective on patient prognosis, and guide the surgeon in choosing the ideal therapeutic approach.

## Figures and Tables

**Table 1:** Patient characteristics.

Patient Characteristics	N	%
Sex		
Male	36	65
Female	19	35
Age		
Min	41	
Median	58	
Max	80	
Presurgical radiotherapy		
Yes	30	55
No	25	45
Presurgical chemotherapy		
Yes	50	91
No	5	9
Pre-stenting dysphagia		
Yes	19	35
No	36	65
Comorbidities		
Yes	45	82
No	10	18
Diabetes mellitus		
Yes	14	25
No	41	75
Hypertension		
Yes	34	62
No	21	38
Hypoalbuminemia		
Yes	17	31
No	38	69
Anemia		
Yes	33	60
No	22	40
Hyperglycemia		
Yes	8	15
No	47	85
Smoker		
Yes	40	73
No	15	27
Alcohol consumption		
Yes	33	60
No	22	40
BMI [kg/m^2^]		
Min	19.6	
Median	22.7	
Max	27.7	
Tumor type		
Adenocarcinoma	46	84
Squamous cell carcinoma	9	16
Cancer stage		
II	17	31
III	34	62
IV	4	7
Tumor diameter (cm)		
Min	2	
Median	3	
Max	4	

**Table 2:** Surgical outcomes.

Surgical Outcomes	n	%
Surgery technique		
Ivor Lewis (Eso-gastric)	17	29
McKeown (Eso-gastric cervical)	4	7
Gastrectomy with esophagojejunostomy (Eso-jejunal)	34	64
Fistula size (mm)		
Min	6	
Median	8	
Max	20	
Fistula location		
Eso gastric	17	31
Eso-gastric cervical	4	7
Eso-jejunal	34	62
Stent size (mm)		
16/100	4	7
22/120	5	9
24/120	32	58
36/140	14	25
Mediastinitis		
Yes	12	22
No	43	78
Postprocedural survival		
Yes	52	95
No	3	5
Fistula closure rate	54	98
After first stent	50	91
After second stent	4	7
Average hospital stay by anastomosis (days)	11.7	
Eso-gastric	19.1	
Eso-gastric cervical	4.5	
Eso-jejunal	8.9	

## Data Availability

The data presented in this study are available on request from the corresponding author due to privacy reasons.
